# The Development of New Species-Specific Molecular Markers Based on 5S rDNA in *Elaeagnus* L. Species

**DOI:** 10.3390/plants10122713

**Published:** 2021-12-10

**Authors:** Oleg S. Alexandrov, Gennady I. Karlov

**Affiliations:** All-Russia Research Institute of Agricultural Biotechnology, Timiryazevskaya 42, 127550 Moscow, Russia; karlov@iab.ac.ru

**Keywords:** *Elaeagnus* L. species, 5S rDNA non-transcribed spacers, DNA polymorphism, molecular markers, species identification

## Abstract

The *Elaeagnus* L. species are trees and bushes that mainly grow in temperate zones of Western Europe; Minor, Central, and Southeast Asia; the Far East; and North America. Some species are used as fruit or ornamental plants and have economic value. Problems with the identification of species in the *Elaeagnus* genus by molecular genetical methods arise in the study of populations, systematics, breeding, and other areas of plant science and practice. Recently, the polymorphism of 5S ribosomal DNA non-transcribed spacers (5S rDNA NTSs) in Elaeagnaceae Adans. has been described. The results were used in our study as a basis for development of new species-specific molecular markers for some members of the *Elaeagnus* genus. The author’s method was applied for finding regions that were potentially applicable for species-specific primer design. As a result, some species-specific molecular markers were developed for *Elaeagnus angustifolia* L., *E. commutata* Bernh., *E. pungens* Thunb., and *E. multiflora* Thunb. These markers were tested in a range of samples and showed the presence of amplified fragments in lanes of the marked species only. Samples of other species showed no amplifications. Thus, the developed markers may be useful for the species identification of the studied *Elaeagnus* plants in botanical, dendrological, and genetic research (especially in a leafless period of year), as well as in breeding and hybridization experiments.

## 1. Introduction

The Elaeagnaceae Adans. family consists of three genera: *Elaeagnus* L., *Hippophae* L., and *Shepherdia* Nutt. [1]. In terms of the number of species, *Elaeagnus* is superior to the rest. It comprises nearly 100 species, which are trees and bushes that mainly grow in temperate zones of Western Europe; Minor, Central, and Southeast Asia; the Far East; and North America. These plants have shoots and leaves that are tightly covered with scales or hairs, and therefore the plants appear to have a silvery green color. The *Elaeagnus* species are phanerophytes with shoot tips and buds which are located high above the soil surface and lack protection during unfavorable seasons [2]. In addition, many of the species can grow in regions with poor soil, due to their symbiosis with nitrogen-fixing bacteria living in their root nodules. [3]. Such species as *Elaeagnus angustifolia* L., *E. commutata* Bernh., *E. pungens* Thunb., *E. umbellata* Thunb., and *E. multiflora* Thunb. are used as fruit or ornamental plants and have economic value. The fruit of *E. angustifolia*, *E. multiflora*, and *E. umbellata* are widely used for food [4]. In different countries, breeding of these species is underway. Varieties of these *Elaeaegnus* species, as well as *E.* × *ebbingei* Boom. (a hybrid between *E. macrophylla* Thunb. and *E. pungens*), are widely sold and grown. Associated with this, genetic investigations and the development of molecular approaches in *Elaeagnus* species identification are especially needed.

Genetically, the *Elaeagnus* genus is actively studied. The complete genome of *E. angustifolia*, as well as the *E. macrophylla* and *E. conferta* Roxb. chloroplast genomes, has been recently sequenced [5,6,7]. Furthermore, several attempts have been made to identify species in the *Elaeagnus* by using DNA barcoding when determining phylogenetic relationships and describing new species [8,9,10,11]. In addition, different types of molecular markers have been developed to study genetic diversity in *Elaeagnus* populations [12,13,14,15,16]. However, there is no information about the successful development of species-specific molecular markers for such important members of this genus as *E. angustifolia*, *E. commutata*, *E. pungens*, *E. umbellata*, and *E. multiflora*.

Recently, Alexandrov et al. conducted a comparative study of 5S rDNA non-transcribed spacers (5S rDNA NTSs) in the Elaeagnaceae species [17]. Moreover, 5S rRNA genes are organized as tandemly repeated DNA with monomers that consist of a conservative 120 bp coding part and a variable non-coding part (NTS) [18,19]. It was revealed that NTSs often show species-specific polymorphisms [20,21]. The authors successfully used such polymorphisms to develop species-specific molecular markers in a range of plants [22,23,24,25]. In some members of the *Elaeagnus* genus, 5S rDNA NTSs were sequenced and compared. The observed level of interspecies polymorphism among the studied *Elaeagnus* spp. was promising enough for the development of species-specific DNA markers. However, the development of such markers requires searching for specific marker regions within sequences. Alexandrov and Karlov designed species-specific primers for the *Populus* genus, using an effective approach to assess polymorphism between sliding decinucleotide fragments [24]. The approach made it possible to quickly develop species-specific markers for poplars such as *P. nigra* L., *P. deltoides* Bartr. ex Marshall., *P. alba* L., *P. tremula* L., *P. bolleana* Lauche, and some of their hybrids.

In this study, the approach was successfully applied to create some species-specific molecular markers for *E. angustifolia*, *E. commutata*, *E. pungens,* and *E. multiflora*; the markers were tested in a range of samples. They may be useful for species identification of the studied *Elaeagnus* plants in botanical, dendrological, and genetic research (especially in a leafless period of year), as well as in breeding and hybridization experiments.

## 2. Results

### 2.1. Alignments of 5S rDNA Non-Transcribed Spacers (NTSs) among the Elaeagnus Species and Development of Species-Specific Primers

Twelve alignments of *E. angustifolia*, *E. commutata*, *E. pungens,* and *E. multiflora* NTSs were obtained for the search of high polymorphic decinucleotide fragments, which could be a basis for species-specific marker development (Supplementary Materials Tables S1–S12).

In the case of *E. angustifolia* specific marker, the MW288958 NTS of this species was aligned with MW288964–MW288968 NTSs of *E. commutata* (Supplementary Materials Table S1), MW288974–MW288976 NTSs of *E. pungens* (Supplementary Materials Table S2), and MW288969–MW288973 NTSs of *E. multiflora* (Supplementary Materials Table S3). The first alignment consisted of 210 columns and showed a level of decinucleotide fragment polymorphism in the range of 10–74%. The comparative analysis of values at points of the alignment revealed two fragments (39th and 163rd; the numbering of decinucleotide fragments starts from the tenth column of the alignment) with a maximal level of polymorphism at different ends of the MW288958 sequence, i.e., 70% and 74%, respectively. The 14–20 bp regions containing these fragments were tested by using a multiple primer analyzer. As a result, the regions containing the 35th–45th (correspond to the forward primer Elang1f) and 162nd–175th (correspond to the reverse primer Elang1r) fragments were chosen for further testing (in Supplementary Materials Table S1 and in other alignments, the chosen regions are indicated by red letters). The sequences of these fragments were detected in two other alignments (Supplementary Materials Tables S2 and S3). In the alignment with *E. pungens* NTSs, these regions had polymorphism levels in the ranges of 37–47% (the first region, corresponding to the forward primer) and 30–60% (the second region, corresponding to the reverse primer). In the alignment with NTSs of E. multiflora, the polymorphism levels in the studied regions were in the ranges of 30–60% and 34–70%, respectively. Although the polymorphism levels of the studied regions in the second and the third alignments were not maximum, they were high enough, and sequences of these regions were used for the synthesis of primers and PCR.

The *E. commutata* specific marker was developed according to the following scenario. The MW288964 NTS sequence of *E. commutata* was aligned with the MW288958–MW288963 NTSs of *E. angustifolia* (Supplementary Materials Table S4), MW288974–MW288976 NTSs of *E. pungens* (Supplementary Materials Table S5), and MW288969–MW288973 NTSs of *E. multiflora* (Supplementary Materials Table S6). The fourth alignment consisted of 210 columns. It showed a 10–80% level of decinucleotide fragment polymorphism. The comparative analysis of values at points of the alignment and combination self-dimer/cross-dimer free regions led to the selection of four variants. The following regions were chosen for further verification: (1) containing 7th–24th fragments (with a 45–60% level of decinucleotide fragment polymorphism, Elcom2-f primer), (2) containing 28th–41st fragments (with 17–35% level of this indicator, Elcom1-f primer), (3) containing 157th–169th fragments (with 38–60% level of this indicator, Elcom1-r primer), and (4) containing 170th–182nd fragments (with 48–80% level of this indicator, Elcom2-r primer). The sequences of these fragments were detected in the alignments with *E. pungens* and *E. multiflora* NTSs (Supplementary Materials Tables S5 and S6). The levels of decinucleotide fragment polymorphism in the correspondent regions of these alignments were determined to be suitable for further PCR testing.

In the *E. pungens* specific marker development, MW288969 NTS of this species was aligned with the MW288958–MW288963 NTSs of *E. angustifolia* (Supplementary Materials Table S7), MW288964–MW288968 NTSs of *E. commutata* (Supplementary Materials Table S8), and MW288969–MW288973 NTSs of *E. multiflora* (Supplementary Materials Table S9). The analysis of the seventh and the eighth alignments revealed a range of regions with high values of polymorphism (about 60–70%; see graphs in the corresponding tables), but they were not optimal in the ninth alignment, because the level of polymorphism between *E. pungens* and *E. multiflora* NTSs was not high. However, a short region near the end of this alignment (195th–200th columns) might be perspective 3′ end specific primers, according to the author’s opinion. Thus, the Elpung1-f forward primer containing the 179th–190th fragments was selected. The reverse primer was designed on the basis of 5S coding sequence. The sequence of the reverse primer was chosen according to the results of self- and cross-dimer checking by the multiple primer analyzer.

The *E. multiflora* specific marker development (in principle, it is possible to talk about the development of *E. multiflora*/*E. umbellata* marker, since their NTSs are similar, as in varieties of the same species [17]) were carried out on the basis of the alignments among MW288969 NTS and MW288958–MW288963 NTSs of *E. angustifolia* (Supplementary Materials Table S10), MW288964–MW288968 NTSs of *E. commutata* (Table S11), and MW288974–MW288976 NTSs of *E. pungens* (Supplementary Materials Table S12). As in the case of the *E. pungens* specific marker development, the level of decinucleotide fragment polymorphism was not high between *E. pungens* and *E. multiflora* NTSs (see Supplementary Materials Table S12). The short region (corresponding to the 195th–200th columns in the ninth alignment) was used in the Elmult1-f forward design as a 3′ end primer (containing the 179th–190th fragments). Three variants of the reverse primer (Elmult1-r, Elmult2-r, and Elmult3-r) were designed on the basis of the 146th–164th fragments. The variants differ in the added starting and ending nucleotides. Since the 146th–164th fragments are situated upstream to the forward primer location, the reverse primers are oriented to the alignment start. This orientation results in amplification of the product that contains the entire 5S coding sequence between adjacent NTSs. Additionally, the Elmult2-f forward primer (containing the 72nd–81st fragments) was designed. All primer pairs were tested by using the multiple primer analyzer.

Thus, several approaches for the development of species-specific primers based on NTS have been successfully applied in different cases with the *Elaeagnus* spp. and have led to the creation of markers that require practical testing during PCR experiments.

### 2.2. PCR Test with Developed Primers

**Figure 1:**
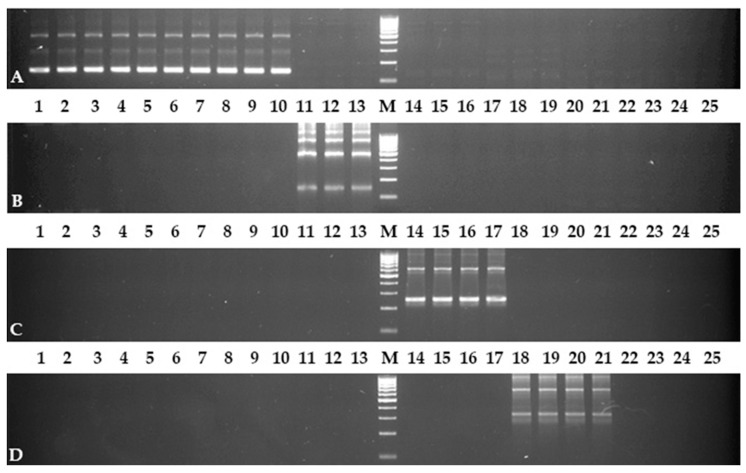
Detection of PCR products by electrophoresis (**A**) with Elang1-f/Elang1-r primers, (**B**) with Elcom1-f/Elcom1-r primers, (**C**) with Elpung1-f/Elpung1-r primers, and (**D**) with Elmult1-f/Elmult2-r primers. Numbers of lanes correspond to numbers of samples in Supplementary Materials Table S13. M, marker of molecular weight with 100 bp stepFinally, different combinations of Elmult1-f and Elmult2-f, as well as Elmult1-r, Elmult2-r, and Elmult3-r, were tested by using a PCR experiment (the tests were carried out among a limited number of samples selected according to the principle “one species–one sample” for *Elaeagnus* spp. and *H. rhamnoides* as a control). The Elmult1-f/Emult3-r pair did not show the amplified fragments in all samples and was dismissed as a very unfortunate pair (Figure S1). All remaining pairs showed target amplification in the *E. multiflora* and *E. umbellata* samples (the NTSs of these species look similar to NTSs of one species). However, the Emult2-f/Emult1-r and Emult2-f/Emult2-r pairs also demonstrated the amplified fragments in *E. pungens* sample. Thus, the best pairs were Elmult1-f/Elmult1-r, Elmult1-f/Elmult2-r, and Elmult2-f/Elmult3-r, because they did not have a non-specific amplification in any other *Elaeagnus* species and *H. rhamnoides*. The amplifications with the Elmult1-f/Elmult2-r pair were the brightest among all *E. multiflora* primer combinations (Figure S1). It is likely that the PCR conditions for this pair turned out to be the most optimal. This pair should be recommended first. It was used to check all Elaeagnaceae samples collected for this study (Figure 1D).

All the designed primers were synthesized and used in the PCR experiments. The Elang1-f/Elang1-r primer pair showed the required result. All samples of *E. angustifolia* had fragments of PCR products in the corresponding lanes of electrophoresis gel (Figure 1A). The pattern of the amplified fragments was ladder-like with a step equal to the length of the monomer (coding part 5S + NTS, the similar patterns were also observed with other markers, see Figure 1B–D). The lanes of other *Elaeagnus* species, as well as other Elaeagnaceae members, did not have the amplified fragments at all.

In contrast to the first marker, the Elcom1-f/Elcom1-r primer pair did not show a good result in the same PCR conditions. All *E. commutata* samples had the required fragments, but there was a non-specific amplification in other *Elaeagnus* species, as well as other Elaeagnaceae members. However, the problem was eliminated by increasing the annealing temperature of the primers to 62 °C. After this optimization, an electrophoretic picture, similar to the previous marker, was observed; only samples of *E. commutata* had amplified fragments (Figure 1B). The same results were obtained by using the Elcom2-f/Elcom2-r primer pair.

The PCR experiment with the Elpun1-f/Elpun1-r primer pair was successful the first time. The pattern of the amplified fragments was observed in the *E. pungens* lane, as well as in lanes of all *E.* × *ebbingei* samples (Figure 1C). Since *E.* × *ebbingei* is a hybrid bred with the participation of *E. pungens*, such a result of the Elpun1-f/Elpun1-r marker work was expected. Other lanes (even corresponding to *E. multiflora* samples with NTSs that are very similar to *E. pungens*) were without PCR fragments.

## 3. Discussion

In this study, sequences of *Elaeagnus* spp. NTSs were used to develop species-specific molecular markers. These sequences were obtained by the authors in the course of a previous investigation [17]. Then, the polymorphism of these sequences was assessed as a whole, because the goal of finding their most polymorphic parts was not set. However, creating molecular marker requires such polymorphic regions, and, therefore, decinucleotide fragments were studied in the alignments of NTSs for four *Elaeagnus* spp. This approach has been successfully used in similar investigations, in which species-specific markers in the *Populus* genus were developed [24,25].

As expected, for the decinucleotide fragments in the *Elaeagnus* spp., the NTS alignments showed levels of polymorphism that often differed both from the average over the entire alignment and among themselves. For example, the alignment between MW288958 NTS of *E. angustifolia* and MW288964–MW288968 NTSs of *E. commutata* showed a 36–39% level of polymorphism as a whole (see Supplementary Materials Table S5 in Reference [17]; the level of polymorphism was equal to 100%—values of identity). At the same time, the level of decinucleotide fragment polymorphism was within 10–74% (see graph in Supplementary Materials Table S1 of this article). That is, this alignment includes both more polymorphic and less polymorphic regions as compared with the level of polymorphism in general. In terms of creating molecular markers, the decinucleotide fragments with high levels of polymorphism are interesting in the first place. In simple cases, such fragments themselves become a good basis for the design of species-specific primers. Such a case can be seen in the example of the development of a specific marker for *E. angustifolia*. Two regions at different ends of the alignment with maximal levels of polymorphism were selected for the Elang1-f and Elang1-r primer design, and the primers immediately showed the desired result during the PCR testing.

However, not every fragment can become the basis for designing primers, even if they show a high level of polymorphism. The sequence of nucleotides within the fragment may play an important role, and therefore self- and cross-dimer checking is necessary. Thus, fragments with non-maximal levels of polymorphism were also selected. For example, the development of *E. commutata* specific marker includes the fragments with a 17–35% level of polymorphism (primer Elcom1-f). The PCR test with this primer revealed non-specific amplification in other *Elaeagnus* species, as well as other Elaeagnaceae members, under conditions similar to those with the *E. angustifolia* specific marker. Optimization of the PCR condition (increasing the primer annealing temperature to 62 °C) was required to obtain the absence of amplification in all samples, except for the *E. commutata* samples. This case confirmed the conclusions that the stringency of primer annealing is improved and more specific amplification is observed when the PCR annealing temperature is increased [26,27].

The development of *E. pungens* and *E. multiflora* specific markers was associated with some problems, since the polymorphism level of their NTSs was not high (see graphs in Supplementary Materials Tables S9 and S12). However, the most polymorphic region in these NTSs (the 179th–190th fragments) was sufficient for the design of the required markers. Since this region was small, the design in the case with the *E. pungens* specific marker was carried out according to the following strategy (this strategy was recently used to develop species-specific markers for poplar of the *Aigeiros* Daby section [17]). The polymorphic part of this region was used as the 3′ end of the forward primer (Elpung1-f), and the reverse primer was designed on a basis of the 5S coding sequence (Elpung1-r). Thus, the successful work of this primers confirmed the effectiveness of this strategy for the second time.

The *E.* × *ebbingei* samples showed a target amplification with the Elpung1-f/Elpung1-r primer pair similar to the *E. pungens* sample. This fact was quite expected, because *E. pungens* is one of the parents for *E.* × *ebbingei*. Hybrids include NTSs of both parents, and the NTS-based species-specific markers of both parents work with the DNA matrix of these hybrids. The same results have been described for hybrid poplars, such as *P. × canadensis* Moench. [24] and *P. × canescens* (Aiton) Sm. [25]. Unfortunately, the case with *E.* × *ebbingei* cannot yet be distinguished from *E. pungens* by the Elpung1-f/Elpung1-r marker, because the NTS of the second parent (*E. macrophylla*) was not sequenced, and there is no marker for this species. The authors expect that this theme will be a subject of further investigation, since *E.* × *ebbingei* plants have conspicuous value as an ornamental plant.

The previously described 179th–190th fragments were also used in the *E. multiflora* specific marker development (Supplementary Materials Table S12). In this development, another strategy was used. The twelfth alignment contained another polymorphic region (the 146th–164th fragments), which is located near the 179th–190th fragments. To obtain the PCR product with conveniently detectable length, a direction of the primers (which were based on these regions) was changed. The downstream located region was used for the forward primer (Elmult1-f) and the upstream located region was used for the reverse primers (Elmult1-r, Elmult2-r, and Elmult3-r). As a result, the PCR products were obtained when the primers annealed to adjacent monomers. These PCR products included the entire 5S cording sequence. In addition to this strategy, the classical approach (as in the *E. angustifolia* specific marker development) was also applied for the *E. multiflora* specific marker development, and the upstream located forward primer was designed (Elmult2-f). The PCR testing revealed that both approaches resulted in success (Elmult1-f/Elmult1-r, Elmult1-f/Elmult2-r, and Elmult2-f/Elmult3-r pairs allowed us to obtain the required amplification pictures). When discussing the *E. multiflora* specific marker, it is worthwhile paying attention to the different effects of Elmult1-r, Elmult2-r, and Elmult3-r used in the different combinations. These reverse primers are almost identical, having only 1–2 bp differences at their 5′ and 3′ ends. However, the Elmult3-r with the Elmult-1f resulted in the absence of amplification in all samples, while the Elmult2-f/Elmult3-r pair worked appropriately. The “Elmult2-f/Elmult1-r”–“Elmult1-f/Elmult1-r” and “Elmult2-f/Elmult2-r”–“Elmult1-f/Elmult2-r” pairs showed some differences in the PCR results. Thus, it was confirmed that the 1–2 bp differences at the 5′ and 3′ ends may play a key role in the NTS-based species-specific marker development.

The PCR results with the *E. multiflora* specific marker revealed that *E. umbellata* samples also had PCR products. Such a picture was expected, because the NTSs of these species looked similar to the NTSs of one species. They showed a level of polymorphism in the range of 3–7% (see Supplementary Materials Table S5 in Reference [17], the levels of polymorphism are equal to 100%—values of identity). There is an opinion that these two species are one species [28]. In such situation, they may have some genetic differences that are not detectible by the NTS-based species-specific markers. It is possible that other types of molecular markers can reveal these differences. The development and practical studying of such markers is a perspective for future research to continue the theme of this study.

In summary, a system of species-specific molecular markers for *E. angustifolia*, *E. commutata*, *E. pungens*, and *E. multiflora* identification was created and tested. The markers may be recommended as a tool for molecular species identification of the studied *Elaeagnus* plants in botanical, dendrological, and genetic research. The application of these markers will be especially useful in a leafless period of the year. Moreover, they may be applied in breeding and hybridization experiments to confirm the hybrid nature of the obtained plants.

## 4. Materials and Methods

### 4.1. Plant Material and DNA Isolation

In this study, a collection of *Elaeagnus* spp. samples was created (Supplementary Materials Table S13). The *E. angustifolia*; *E. commutata*; *E. multiflora; E.* × *ebbingei* var. “Lime light” and “Gilt Edge”; and *E. umbellata* var. “Pointilla Sweet’n’Sour”, “Pointilla Fortunella”, and “Pointilla Amoroso” plants were grown in open ground in KIZ “Allea”, Kievsky village, Moscow, Russia. The *E.* × *ebbingei* var. “Compacta” bush was grown in open ground near Dmitrovskoe shosse 124A, Moscow, Russia. The sample of *E. pungens* var. “Maculata” was grown in a greenhouse at the All-Russia Research Institute of Agricultural Biotechnology, Timiryazevskaya 42, Moscow, Russia. The leaf-tissue samples of *Sh. argentea* and *Sh. canadensis* were received as dried material from the Arnold Arboretum of Harvard University (Boston, MA, USA) and the Botanic Garden Meise (Meise, Belgium), respectively. The leaf-tissue samples of *H. rhamnoides* and *H. salicifolia* were received as dried material from the Jardin botanique de Lyon (France) and the Rogów Arboretum of Warsaw University of Life Sciences (Poland), respectively. The DNA isolation was carried out according to the Doyle and Doyle CTAB protocol with some modifications [29,30]. The DNA samples were equalized in concentration, aliquoted, and stored at –20 °C.

### 4.2. Analysis of Sequences and Primer Design

Nineteen NTS 5S rDNA *Elaeagnus* spp. sequences were collected from the GenBank (*E. angustifolia,* MW288958–MW288963; *E. commutata,* MW288964–MW288968; *E. pungens,* MW288974–MW288976; *E. multiflora,* MW288969–MW288973). All alignments of sequences were carried out by using the GenDoc software [31]. The processing of the polymorphism analysis in 10-column fragments was as follows. In the alignment, one sequence of the marked species (MW288958 for *E. angustifolia*, MW288964 for *E. commutata,* MW288974 for *E. pungens*, and MW288969 for *E. multiflora*) was used with all sequences of another species. Thus, three alignments were obtained for each marker. The alignments were all divided into ten-column fragments (the first column of each fragment was the second column of the previous fragment). The level of polymorphism in each fragment was calculated as the ratio of the number of polymorphic sites (nucleotides that differ from the nucleotides of the studied sequence or gaps) to the total number of sites in the fragment among all the sequences of another species. The neighboring fragments with a high level of polymorphism were used for the primer design. All the designed primer pairs were checked with a multiple primer analyzer (Thermo Fisher Scientific, Waltham, MA, USA; see link in Supplementary Material S15) to avoid self-dimers and cross-dimer free primers (Table 1).

The designed primers were synthesized by ZAO “Evrogen” (Moscow, Russia) and ZAO “Syntol” (Moscow, Russia).

### 4.3. The PCR Experiments and Electrophoresis

PCR was carried out under the following conditions: 94 °C for 3 min, 35 cycles of 94 °C for 30 s, N °C for 30 s, 72 °C for 1 min, and 72 °C for 10 min. The PCR mix included approximately 10 ng of genomic DNA, 2.5 U of Taq-polymerase (ZAO “Evrogen”, Moscow, Russia), 1× Taq Turbo-buffer, 2.5 mM of MgCl_2_, 100 µM of each dNTP, and 0.25 µM of the forward and reverse primers and ddH_2_O. The PCR products were detected by electrophoresis on 2.5% agarose gel at 10 V/cm in 0.5 M of TBE buffer by using a Sub-Cell Model 192 camera (Bio-Rad, Hercules, CA, USA) and photographed by using the gel documentation system GelDoc XR Plus (Bio-Rad, Hercules, CA, USA).

## Figures and Tables

**Table 1:** Designed primers and their parameters.

Primer Name	Sequence	Annealing, t °C ^1^	PCR Product Length, bp
Elang1-f	5′-TCGATCAACCGAATCAAACA-3′	59.0	150
Elang1-r	5′-CGAAACTTGTTATTTTTGCGAAT-3′		
Elcom1-f	5′-TCTAATCCGATAAACCGAATTGT-3′	62.0	151
Elcom1-r	5′-AAAGTATTTCATGCGTGCGTAA-3′		
Elcom2-f	5′-GCTCTATTTTATTCTAATCCGAT-3′	62.0	165
Elcom2-r	5′-GATTTGTACGGGTAAAGTATTT-3′		
Elpung1-f	5′-GTGTAAGTAGAAAGTTGGAAAC-3′	54.0	253
Elpung1-r	5′-AACTCTTCTTATGATTTGGTT-3′		
Elmult1-f	5′-GGATGGGTGACCTCCGG-3′	55.0	328
Elmul1-r	5′-AACGTTCTACATGCATTCGT-3′		
Elmult1-f	5′-GGATGGGTGACCTCCGG-3′	55.0	328
Elmult2-r	5′-GGCGACCCTGGGAAGTGT-3′		
Elmult1-f	5′-GGATGGGTGACCTCCGG-3′	55.0	329
Elmult3-r	5′-TAACGTTCTACATGCATTCG-3′		
Elmult2-f	5′-GCTACATCATCAGTCCAACA-3′	55.0	103
Elmult1-r	5′-AACGTTCTACATGCATTCGT-3′		
Elmult2-f	5′-GCTACATCATCAGTCCAACA-3′	55.0	103
Elmult2-r	5′-GGCGACCCTGGGAAGTGT-3′		
Elmult2-f	5′-GCTACATCATCAGTCCAACA-3′	55.0	104
Elmult3-r	5′-TAACGTTCTACATGCATTCG-3′		

^1^ This is an “N” in Section 4.3.

## Data Availability

*Shepherdia* spp. and *Hippophae* spp. samples were used in this study. The *Sh. argentea* dried leaves were kindly provided by Dr. Kathryn Richardson (AA#102-77*A sample of Arnold Arboretum, The Harvard University, USA). The *Sh. canadensis* dried leaves were kindly provided by Dr. Kenneth Bauters (*19801643-I34ZZ sample of Botanic Garden Meise, Belgium). The *H. rhamnoides* dried leaves were kindly provided by Jean-François Thomas (Jardin botanique de Lyon, France). The *H. salicifolia* dried shoots were kindly provided by Piotr Banaszczak (Rogów Arboretum of Warsaw University of Life Sciences, Poland).

## Supplementary Materials

The following are available online at https://www.mdpi.com/article/10.3390/plants10122713/s1. Table S1: The alignment of *E. angustifolia* NTS (MW288958) with *E. commutata* NTSs (MW288964–MW288968) and graph of the decinucleotide fragment polymorphism values. Table S2: The alignment of *E. angustifolia* NTS (MW288958) with *E. pungens* NTSs (MW288974–MW288976) and graph of the decinucleotide fragment polymorphism values. Table S3: The alignment of *E. angustifolia* NTS (MW288958) with *E. multiflora* NTSs (MW288969–MW288973) and graph of the decinucleotide fragment polymorphism values. Table S4: The alignments of *E. commutata* NTS (MW288964) with *E. angustifolia* NTSs (MW288958–MW288963) and graph of the decinucleotide fragment polymorphism values. Table S5: The alignments of *E. commutata* NTS (MW288964) with *E. pungens* NTSs (MW288974–MW288976) and graph of the decinucleotide fragment polymorphism values. Table S6: The alignments of *E. commutata* NTS (MW288964) with *E. multiflora* NTSs (MW288969–MW288973) and graph of the decinucleotide fragment polymorphism values. Table S7: The alignment of *E. pungens* NTS (MW288969) with *E. angustifolia* NTSs (MW288958–MW288963) and graph of the decinucleotide fragment polymorphism values. Table S8: The alignment of *E. pungens* NTS (MW288969) with *E. commutata* NTSs (MW288964–MW288968) and graph of the decinucleotide fragment polymorphism values. Table S9: The alignment of *E. pungens* NTS (MW288969) with *E. multiflora* NTSs (MW288964–MW288968) and graph of the decinucleotide fragment polymorphism values. Table S10: The alignments of *E. multiflora* NTS (MW288969) with *E. angustifolia* NTSs (MW288958–MW288963) and graph of the decinucleotide fragment polymorphism values. Table S11: The alignments of *E. multiflora* NTS (MW288969) with *E. commutata* NTSs (MW288964–MW288968) and graph of the decinucleotide fragment polymorphism values. Table S12: The alignments of *E. multiflora* NTS (MW288969) with *E. pungens* NTSs (MW288974–MW288976) and graph of the decinucleotide fragment polymorphism values. Table S13: The studied plants of *Elaeagnus*, *Shepherdia*, and *Hippophae* spp. Figure S1: The results of PCR test with different combinations of *E. multiflora* specific primers. Supplemental material S1: The link of the Multiple Primer Analyzer (Thermo Fisher Scientific, Waltham, MA, USA).
