# Supplementary material for: The Development of New Species-Specific Molecular Markers Based on 5S rDNA in Elaeagnus L. Species
Source: Plants (Basel). 2021 Dec 10;10(12):2713. doi: 10.3390/plants10122713 (PMC8704621; doi:10.3390/plants10122713)
Supplement: Supplementary file 1 [file plants-10-02713-s001.zip › Figure S1.pdf]

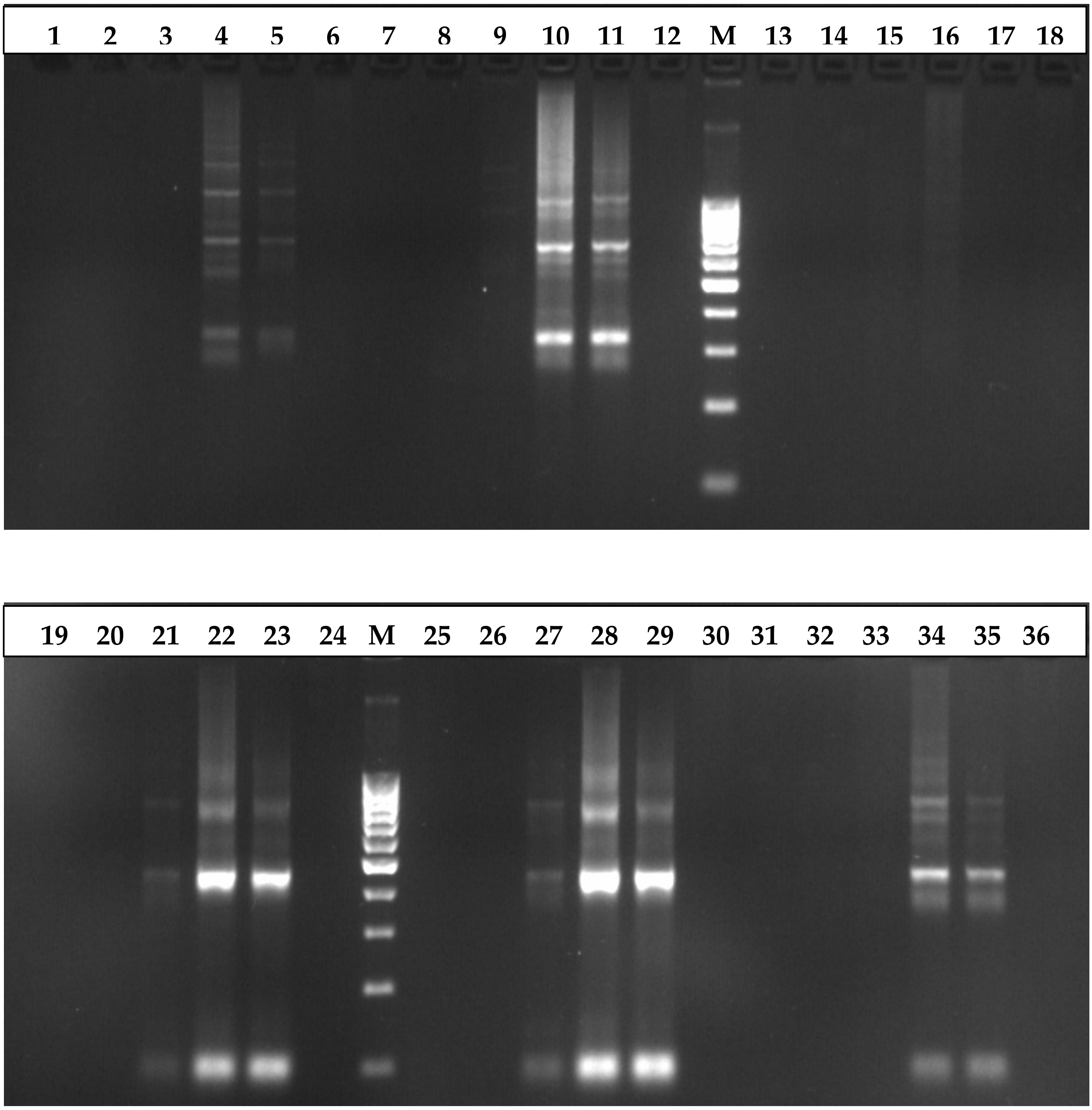

Figure S1. The results of PCR test with different combinations of *E. multiflora* specific primers. The PCR products were obtained with Elmult1-f/Elmult1-r primers (lanes 1–6); with Elmult1-f/Elmult2-r primers (lanes 7–12); with Elmult1-f/Elmult3-r primers (lanes 13–18); with Elmult2-f/Elmult1-r primers (lanes 19–24); Elmult2-f/Elmult2-r primers (lanes 25–30); Elmult2-f/Elmult3-r primers (lanes 31–36). Numbers of lanes correspond to the following samples: *E. angustifolia* (lanes 1, 7, 13, 19, 31); *E. commutata* (lanes 2, 8, 14, 20, 32); *E. pungens* (lanes 3, 9, 15, 21, 33); *E. multiflora* (lanes 4, 10, 16, 22, 34); *E. umbellata* var. "Pointilla Amoroso" (lanes 5, 11, 17, 23, 35); *H. rhamnoides* (lanes 6, 12, 18, 22, 36). M – marker of molecular weight with 100 bp step.
