# Supplementary material for: The Development of New Species-Specific Molecular Markers Based on 5S rDNA in Elaeagnus L. Species
Source: Plants (Basel). 2021 Dec 10;10(12):2713. doi: 10.3390/plants10122713 (PMC8704621; doi:10.3390/plants10122713)
Supplement: Supplementary file 1 [file plants-10-02713-s001.zip › Table S13.pdf]

Table S13. The studied plants of *Elaeagnus*, *Shepherdia* and *Hippophae* spp.

Number	Species	Variety	Code	Origin	Coordinates or greenhouse/dried material
1	*Elaeagnus angustifolia*			‘’Martin Sad’’ nursery, Mytischi, Russia	55°41’49.65’’ 36°84’83.48’’
2				Kerch’, Crimea	45°29’37.12’’ 36°42’86.36’’
3				Near restaurant “Donskaya kukhnya” Rostovskaya oblast’, Russia	47°18’63.27’’ 39°86’95.20’’
4				Tikhoretsk, Krasnodar krai, Russia	45°83’47.62’’ 40°11’28.65’’
5				Hutor Millerov, Rostovskaya oblast’,Russia	47°61’80.54’’ 40°03’83.92’’
6				Krasyukovskoe rural settlement, Rostovskaya oblast’, Russia	47°61’77.66’’ 40°01’83.85’’
7				Hutor Proletarka, Rostovskaya oblast’, Russia	47°87’99.94’’ 40°20’98.60’’
8				Kamensky district, Rostovskaya oblast’, Russia	48°48’98.32’’ 40°34’47.70’’
9				Hutor Diadin, Voronezhskaya oblast’, Russia	49°83’72.83’’ 40°52’72.58’’
10				Boguchar, Voronezhskaya oblast’, Russia	49°91’91.82’’ 40°53’37.85’’
11	*Elaeagnus commutata*			Street planting in ‘’KIZ Allea’’, Kievsky village, Moscow, Russia	55°41’69.27’’ 36°84’76.71’’
12				Yard of School 2044, Moscow, Russia	55°92’63.12’’ 37°54’25.41’’
13				Yard of house 165D4, Dmitrovskoe shosse, Moscow, Russia	55°92’22.91’’ 37°54’27.00’’
14	*Elaeagnus pungens*	‘’Maculata’’		‘’Opt-khoz’’ nursery, Ozery,	greenhouse

				Russia	
15	*Elaeagnus × ebbingei*	“Compacta”		‘’Opt-khoz’’ nursery, Ozery, Russia	55°92’59.63’’ 37°55’03.87’’
16		“Lime light”		‘’Opt-khoz’’ nursery, Ozery, Russia	55°41’48.64’’ 36°84’81.86’’
17		“Gilt Edge”		‘’Opt-khoz’’ nursery, Ozery, Russia	55°41’48.69’’ 36°84’82.02’’
18	*Elaeagnus multiflora*			‘’Nara-Sad’’ nursery, Naro-Fominsk, Russia	55°41’48.48’’ 36°84’79.92’’
19	*Elaeagnus umbellata*	‘’Pointilla Sweet’n’Sour’’		‘’Darvin’’ nursery, Moscow, Russia	55°41’48.64’’ 36°84’85.38’’
20		‘’Pointilla Fortunella’’		‘’Martin Sad’’ nursery, Mytischi, Russia	55°41’48.35’’ 36°84’84.98’’
21		“Pointilla Amoroso”		‘’Martin Sad’’ nursery, Mytischi, Russia	55°41’49.19’’ 36°84’83.52’’
22	*Shepherdia argentea*		Sharg (AA#102-77*A)	Arnold Arboretum, The Harvard University, USA	dried material
23	*Shepherdia canadensis*		Shcan (*19801643-I34ZZ)	Botanic Garden Meise, Nieuwelaan, 38, 1860 Meise, Belgium	dried material
24	*Hippophae rhamnoides*			Jardin botanique de Lyon, France	dried material
25	*Hippophae salicifolia*			Rogów Arboretum of Warsaw University of Life Sciences, Poland	dried material
